# Application of a Multi-Frequency Electromagnetic Method for Boundary Detection of Isolated Permafrost

**DOI:** 10.3390/s25185907

**Published:** 2025-09-21

**Authors:** Yi Wu, Changlei Dai, Yunhu Shang, Lei Yang, Kai Gao, Wenzhao Xu

**Affiliations:** 1 School of Hydraulic and Electric-Power, Heilongjiang University, Harbin 150080, China; 2232031@s.hlju.edu.cn (Y.W.); 2242126@s.hlju.edu.cn (L.Y.); 2251895@s.hlju.edu.cn (W.X.); 2 International Joint Laboratory of Hydrology and Hydraulic Engineering in Cold Regions of Heilongjiang Province (International Cooperation), Harbin 150080, China; 3 Institutional Center for Shared Technologies and Facilities, Northwest Institute of Eco-Environment and Resources, Chinese Academy of Sciences, Lanzhou 730000, China; 4 State Key Laboratory of Cryospheric Science and Frozen Soil Engineering, Northwest Institute of Eco-Environment and Resources, Chinese Academy of Sciences, Lanzhou 730000, China; gaokai@nieer.ac.cn

**Keywords:** isolated permafrost, multi-frequency optimization, boundary detection, electromagnetic inversion, borehole cross-validation

## Abstract

Isolated permafrost is widely distributed in freeze–thaw transition zones, characterized by blurred boundaries and strong spatial variability. Traditional methods such as drilling and electrical resistivity surveys are often limited in achieving efficient and continuous boundary identification. This study focuses on a typical isolated permafrost region in Northeast China and proposes a boundary detection strategy based on multi-frequency electromagnetic (EM) measurements using the GEM-2 sensor. By designing multiple frequency combinations and applying joint inversion, a boundary identification framework was developed and validated against borehole data. Results show that the multi-frequency joint inversion method improves the spatial identification accuracy of permafrost boundaries compared to traditional point-based techniques. In areas lacking boreholes, the method still demonstrates coherent boundary imaging and strong adaptability to geomorphological conditions. The multi-frequency joint inversion strategy significantly enhances imaging continuity and effectively captures electrical variations in complex freeze–thaw transition zones. Overall, this study establishes a complete non-invasive technical workflow—“acquisition–inversion–validation–imaging”—providing an efficient and scalable tool for engineering site selection, foundation design, and permafrost degradation monitoring. It also offers a methodological paradigm for electromagnetic frequency optimization and subsurface electrical boundary modeling.

## 1. Introduction

Permafrost refers to all types of rocks and soils that contain ice and have ground temperatures at or below 0 °C. In China, permafrost covers approximately 22% of the national land area, ranking third globally after Russia and Canada [1]. While the Qinghai–Tibet Plateau is dominated by continuous permafrost [2], the northeastern region is characterized by scattered, structurally complex island permafrost, particularly in the transitional zone of the Greater and Lesser Khingan Mountains. This type of permafrost is typically warm, with features such as shallow thickness, unstable boundaries, and high sensitivity to climate change [3]. With global warming and increased human activity, the number and extent of island permafrost zones are rising. Consequently, establishing efficient and accurate techniques for identifying permafrost boundaries has become a key issue in responding to climate change and engineering demands.

Permafrost boundary surveys play a critical role in permafrost research, with important implications for engineering design, ecological conservation, and resource development. In the field of engineering construction, such surveys provide essential data on the spatial extent, thickness, and physical-mechanical properties of permafrost. This information supports site selection, structural design, construction processes, and post-construction maintenance. As the stability and bearing capacity of permafrost are crucial to engineering safety, accurate boundary identification enables engineers to incorporate permafrost conditions into the design phase, thereby avoiding potential geohazards. In terms of ecological protection, permafrost constitutes a vital component of the cryosphere, and its changes directly affect ecosystem stability [4]. Monitoring permafrost boundaries helps reveal permafrost dynamics and assess environmental impacts, thus offering scientific guidance for ecological restoration and conservation efforts [5]. In resource development, permafrost regions are rich in natural resources, such as gas hydrates [6]. Boundary investigations contribute to determining the spatial distribution and reserves of such resources, providing a scientific basis for exploration and exploitation.

Traditionally, permafrost research in China has relied primarily on drilling and in situ monitoring, which, while accurate, are limited by high costs and low spatial coverage. As a result, they cannot effectively capture detailed permafrost distribution between boreholes. Geophysical methods such as electrical resistivity sounding, ground-penetrating radar (GPR), and electromagnetic induction have been applied to obtain permafrost distribution data. However, their resolution and depth limitations reduce their effectiveness in boundary identification, especially in complex settings involving high water content and multi-layered transition zones [7,8].

To address these challenges, this study employs electromagnetic survey techniques to explore permafrost boundaries. Multi-frequency electromagnetic methods have become a well-established geophysical approach that enables the acquisition of subsurface conductivity data non-destructively. These methods offer several advantages, including adjustable detection depth, high resolution, fast survey speed, low cost, and adaptability to complex geological conditions [9]. Although non-destructive techniques such as high-density resistivity, GPR, seismic refraction, and reflection methods are commonly used to investigate the upper active layer and temperature profiles of permafrost, precise identification of permafrost boundaries remains lacking.

As a novel electromagnetic induction device, the GEM-2 multi-frequency electromagnetic sensor offers new possibilities for permafrost boundary detection due to its portability, flexibility, and efficiency. The operational principles and technical configuration of the GEM-2 system are detailed in Section 2.2. GEM-2 has been widely applied in subsurface structure identification, shallow geological surveys [10], groundwater exploration [11], soil contamination monitoring [12], as well as cavity detection [13], unexploded ordnance localization [14], engineering geology investigations [15], and archaeological exploration [16]. However, its application in permafrost boundary detection remains largely unexplored. Given the importance of boundary delineation in engineering design, ecological protection, and resource development, further research is urgently needed to realize the potential of the GEM-2 system in this domain. Leveraging its multi-frequency capabilities may overcome the limitations of traditional methods and enable high-precision identification of permafrost boundaries, thereby providing robust technical support for permafrost research and related applications.

To this end, a typical island permafrost region in the Greater Khingan Mountains was selected as the study area. A detailed description of the study area is provided in Section 2.1. Using the GEM-2 multi-frequency electromagnetic sensor, this study conducted electromagnetic measurements of island permafrost boundaries, validated the results with borehole data, and developed a multi-frequency inversion and boundary recognition method. The study also examined the imaging characteristics and detection performance of different frequency combinations in response to sharp conductivity transitions. The results not only address a technical gap in high-precision permafrost boundary identification but also provide empirical support for the application of multi-frequency electromagnetic methods in permafrost regions. This research offers scientific guidance for future permafrost surveys, engineering site selection, and foundation design, and holds significant implications for advancing permafrost boundary detection.

## 2. Materials and Methods

### 2.1. Study Area

The Daxing’anling region is located in the northwestern part of Heilongjiang Province, China, spanning across both Heilongjiang and the Inner Mongolia Autonomous Region. The region has an average elevation of approximately 573 m and features a dense network of river systems, including the Heilongjiang (Amur) and Nenjiang river basins. As a key national forest zone, the area boasts a forest coverage rate of 80.95%. Due to its high latitude, inland location, and persistent influence from the Mongolian high-pressure system, the region exhibits a distinct cold temperate continental monsoon climate.

The cold annual mean temperature in this area provides fundamental conditions for the development and maintenance of permafrost, making the Daxing’anling region a prominent southern extension of the Eurasian permafrost boundary [17]. Conducting permafrost boundary detection in this region is of considerable significance for delineating high-latitude permafrost zones in China.

**Figure 1:**
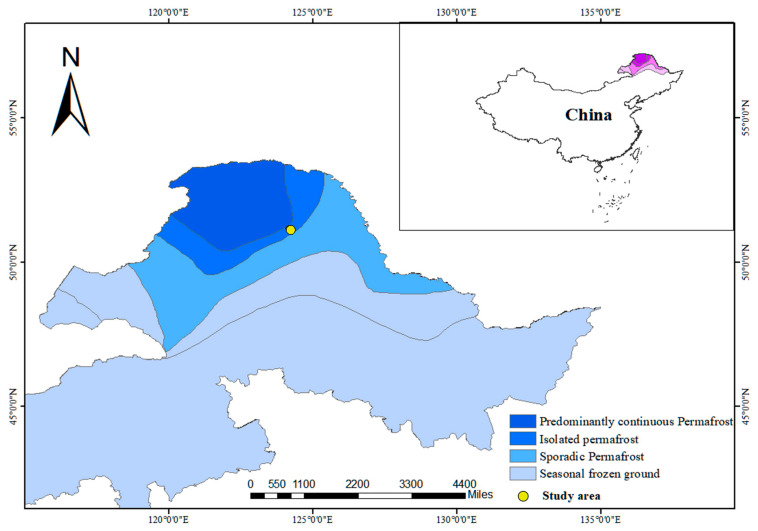
Location of the study area in the northern Greater Khingan Mountains, Northeast China. Schematic background shows generalized permafrost distribution (dark blue: continuous; blue: discontinuous; light blue: seasonal frozen ground). Survey site (51.159° N, 124.186° E) is marked by yellow circle. Inset shows regional context.

As shown in Figure 1, the study site (51.159° N, 124.186° E) is located in the northern Greater Khingan Mountains, within the isolated permafrost zone. Island permafrost in this region is primarily distributed in low-lying areas and sparsely vegetated zones, and is strongly influenced by both topographic and climatic factors. With ongoing global warming, the spatial distribution of permafrost in the Daxing’anling region has become increasingly complex and variable [18].

In this study, we employed the GEM-2 multifrequency electromagnetic instrument developed by Geophex Ltd. (Raleigh, NC, USA) to investigate the permafrost boundaries in the selected study area. The survey was conducted along a roadside transect within a representative island permafrost zone in the Daxing’anling Mountains. The study area is characterized by a cold climate with marked seasonal variations in the thickness of the active layer. Groundwater dynamics and complex thermal–hydrological–salinity coupling exert significant influence on electromagnetic signal responses.

Geomorphologically, the area is dominated by low mountains and rolling hills with gentle relief, forming part of the cold-temperate coniferous forest belt of the Daxing’anling Mountains. This belt represents the southern extension of the Siberian boreal forest zone. Due to climatic constraints, regional vegetation diversity is relatively low. The permafrost type in the study area is classified as high-latitude permafrost, with a mean annual air temperature of approximately −1.2 °C. The climate is defined by long, cold winters and short summers, consistent with the characteristics of a cold temperate continental monsoon climate.

### 2.2. Electromagnetic Principles and Configuration of the GEM-2 Instrument

The GEM-2 is a multi-frequency electromagnetic induction instrument based on the principles of electromagnetic induction. The GEM-2 ground-based system comprises a transmitter unit, receiver unit, compensation coils, a data acquisition and central control module, and auxiliary subsystems including GPS positioning and ambient noise monitoring. The transmitting coil (Tx), compensation coil, and receiving coil (Rx) are configured in a fixed coplanar geometry, with a set coil spacing of 1.66 m [9]. By adjusting the operating frequency, it is possible to target subsurface layers at different depths, thereby enhancing adaptability in complex geologic environments. In multi-layered media, this technique enables the extraction of rich electromagnetic information, supporting detailed structural and compositional analysis of the subsurface.

**Figure 2:**
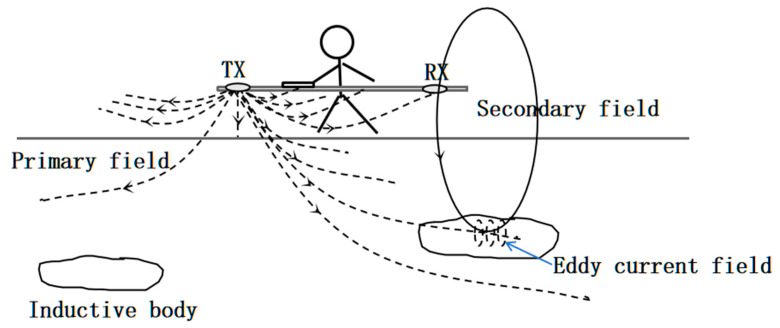
Schematic diagram of the working principle of the GEM-2 multi-frequency electromagnetic induction system. The transmitter coil (Tx) generates a primary electromagnetic field, which induces eddy currents in the subsurface inductive body. These eddy currents produce a secondary magnetic field that is detected by the receiver coil (Rx). A compensation coil is incorporated in the system to suppress the strong primary field and enhance the extraction of the secondary field response, which carries information on the electrical conductivity of the subsurface.

**Figure 3:**
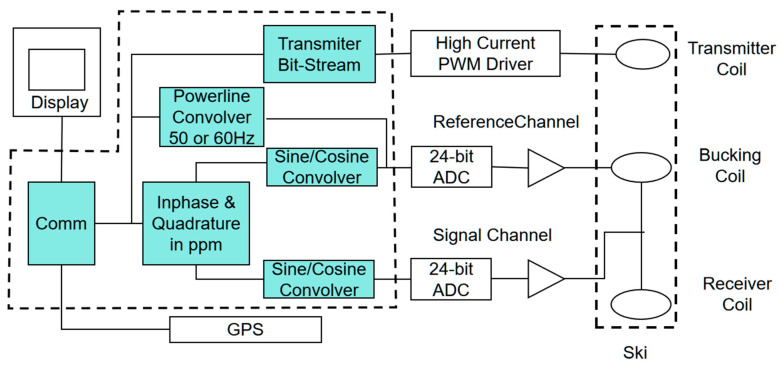
GEM-2 Test Logic Diagram.

The fundamental working principle of the GEM-2 system relies on the electrical and magnetic properties of subsurface media. The transmitter generates a primary low-frequency electromagnetic field composed of multiple discrete frequencies. When this primary field encounters subsurface conductive materials, it induces eddy currents that, in turn, produce a secondary magnetic field. As shown in Figure 2, when the primary field generated by the transmitter coil (Tx) interacts with a conductive subsurface body, eddy currents are induced, producing a secondary magnetic field that is detected by the receiver coil (Rx). The compensation coil facilitates suppression of the primary field, ensuring that the recorded response primarily reflects subsurface conductivity. This secondary field superimposes with the primary field transmitted through the air. The resulting combined signal is then detected and recorded by the receiving coil for further analysis [19]. Figure 3 presents the system block diagram of the GEM-2, which integrates the transmitter unit, receiver unit, bucking coil, signal channels, GPS positioning, and data processing modules. This configuration ensures stable multi-frequency operation and reliable conversion of in-phase/quadrature responses into apparent conductivity values for subsequent inversion.

In GEM-2 electromagnetic sounding technology, the signal received by the receiving coil contains contributions from both the primary and secondary magnetic fields. However, only the secondary field encodes information about the electrical conductivity of the subsurface media. Therefore, during data processing, it is essential to apply signal correction procedures to effectively eliminate the influence of the primary field.

To achieve this, the system utilizes compensation coils to facilitate the separation of the primary and secondary field components. Given that the amplitude of the primary field is typically orders of magnitude greater than that of the secondary field, further enhancement is required to ensure accurate extraction of the secondary signal. This is accomplished by calculating the ratio of the secondary field to the primary field, followed by the application of a normalization procedure [20]. Such normalization enhances the stability and comparability of the conductivity measurements across multiple frequencies and survey conditions. The main technical specifications of the GEM-2 system are summarized in Table 1.

(1) $$H_{\mathrm{abn}}=\frac{H_{S}}{H_{P}}\times 10^{6}$$

In the formulation, $H_{\mathrm{abn}}$ represents the normalized secondary field, also referred to as the relative magnetic anomaly, expressed in parts per million (ppm). The normalized secondary field is a complex quantity and can be expressed as follows:

(2) $$H_{\mathrm{abn}}=I+iQ$$

In the expression, I and Q denote the in-phase and quadrature components of the signal, respectively, and i represents the imaginary unit.

**Table 1:** Technical Specifications of the GEM-2 Multifrequency Electromagnetic Instrument.

Operation Mode	Frequency Domain Instrument
Number of Frequencies	Simultaneous acquisition of up to 10 frequency data points (typically 3–5).
Bandwidth	25 Hz to 96 kHz
Sampling Rate	25 Hz to 30 Hz
Device Dimensions	185 cm × 12.5 cm, 3.6 kg
Coil Parameters	Separated coplanar coils
Power Supply	12V DC
Output	Each frequency is recorded in-phase and quadrature, with power line noise included. Apparent conductivity and susceptibility can be computed.
Coil Configuration	Coplanar (horizontal or vertical)
Coil Separation	1.66 m

### 2.3. Data Acquisition and Field Deployment

During the data acquisition phase, the GEM-2 multifrequency electromagnetic profiler was employed for in situ conductivity measurements. To minimize perturbations caused by terrain-induced antenna motion, survey lines were preferentially laid out over relatively flat and undisturbed surface areas. When necessary, repeated surveys along the same lines were performed to enhance data stability and reproducibility.

The GEM-2 system offers a wide range of operational parameters and supports two primary coil configurations: horizontal coplanar (HCP) and vertical coplanar (VCP) modes. The HCP mode is more widely used in field applications due to its greater effective depth of investigation. The instrument operates within a frequency range of 25 Hz to 93 kHz and supports simultaneous acquisition of multiple frequencies. However, as the total transmitted power is fixed, increasing the number of frequencies leads to a reduced energy allocation per channel, resulting in decreased signal amplitude and consequently lower signal-to-noise ratio (SNR). To optimize measurement depth and data quality, a careful balance must be struck between the number of frequencies used, target depth, signal amplitude, and ambient electromagnetic noise conditions [21]. For most applications, the use of 3 to 5 frequencies is recommended to maintain acceptable SNR and effective resolution.

Frequency selection is a critical aspect of the GEM-2 configuration, as it directly affects both the penetration depth and the spatial resolution of the results. Lower frequencies offer greater skin depth and deeper penetration but produce weaker induced voltages at the receiver coils. Conversely, higher frequencies generate stronger signal amplitudes but are limited in penetration depth. In the context of permafrost detection, this trade-off plays a vital role. The selection of optimal frequency sets must account for the target detection depth, environmental noise, and the desired imaging resolution to ensure accurate and reliable delineation of permafrost boundaries.

(3) $$\delta =\sqrt{\frac{2}{\mu \sigma \omega }}=\sqrt{\frac{2}{\mu \sigma 2\pi f}}$$

In the expression, μ denotes the magnetic permeability of the medium; σ represents the electrical conductivity; *f* indicates the operating frequency

(4) $$DI\approx \alpha \cdot \delta^{\beta }(\alpha \approx 0.94,\beta \approx 0.53)$$

**Figure 4:**
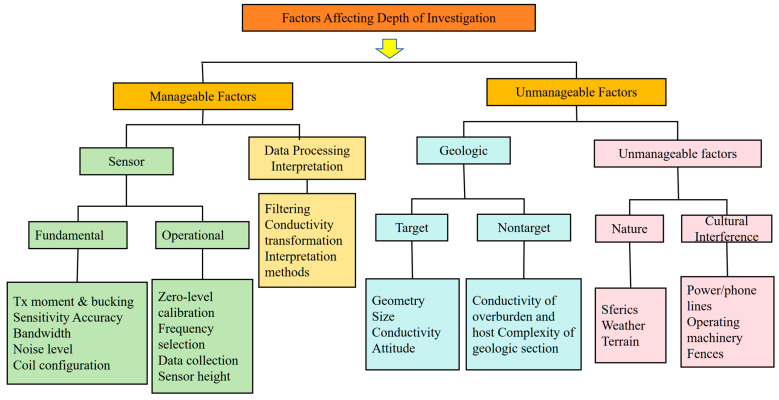
Factors Influencing the Detection Depth of GEM-2 (the category labeled Cultural Interference refers to anthropogenic, non-geological sources of noise that can significantly impact data quality, such as power lines, metal fences, and operating machinery).

For the GEM-2 system, the fixed coil spacing is 1.66 m, and the effective investigation depth can be optimized by employing a combination of multiple frequencies to achieve a complementary “shallow–intermediate–deep” sensing configuration. Low-frequency signals are more suitable for identifying deeper permafrost layers, while high-frequency signals are better suited for resolving the structure of the shallow active layer. In practice, the achievable depth of investigation is influenced not only by frequency configuration but also by a range of manageable and unmanageable factors, as summarized in Figure 4.

**Figure 5:**
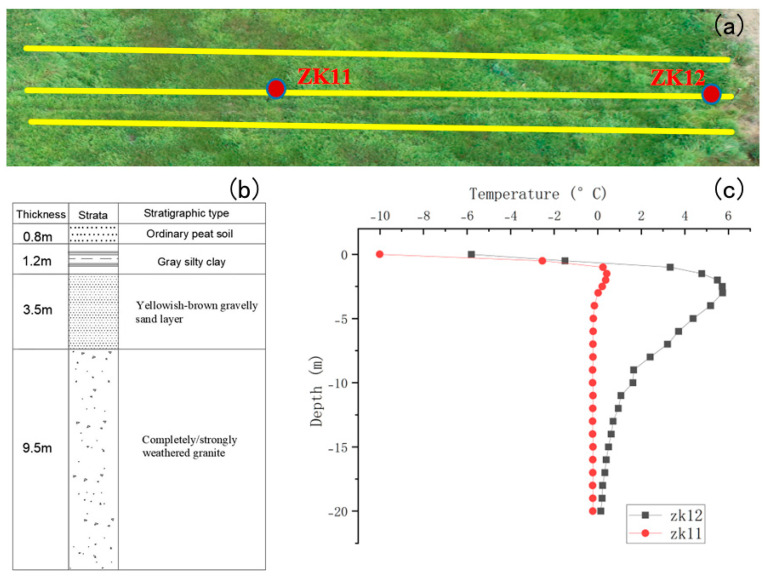
Borehole locations, electrical stratigraphy, and geothermal profiles in the study area. (**a**) UAV orthophoto image of the field site showing the survey lines (yellow lines) and borehole locations (ZK11 and ZK12, red dots). (**b**) Electrical stratigraphic log of borehole ZK11, illustrating the layered resistivity structure of the permafrost zone. (**c**) Geothermal profiles measured at boreholes ZK11 (red circles) and ZK12 (black squares), indicating the temperature-depth relationship in permafrost (ZK11) and non-permafrost (ZK12) settings.

Borehole ZK11, located within the permafrost zone, and ZK12, situated in a non-permafrost area, were selected as control points. As illustrated in Figure 5a, survey lines were deployed along the direction intersecting both boreholes and oriented perpendicular to the anticipated permafrost boundary, in order to maximize sensitivity to electrical conductivity gradients. Based on the spatial characteristics of permafrost distribution in the study area, a line spacing of 1 m was adopted to ensure adequate spatial resolution of the geophysical imaging. Data acquisition was carried out in a continuous walking mode, where the operator walked along each profile while the GEM-2 instrument recorded measurements at a predefined sampling rate, capturing multiple data points per second to ensure acquisition efficiency and continuity. Prior to formal data collection, a preliminary assessment of the surrounding electromagnetic environment was conducted to minimize the influence of potential interference sources such as high-voltage power lines and wireless communication devices, thereby enhancing the accuracy and reliability of the measurements. The inversion strategy and processing workflow are described in Section 2.4.

### 2.4. Inversion Strategy and Processing Workflow

**Figure 6:**
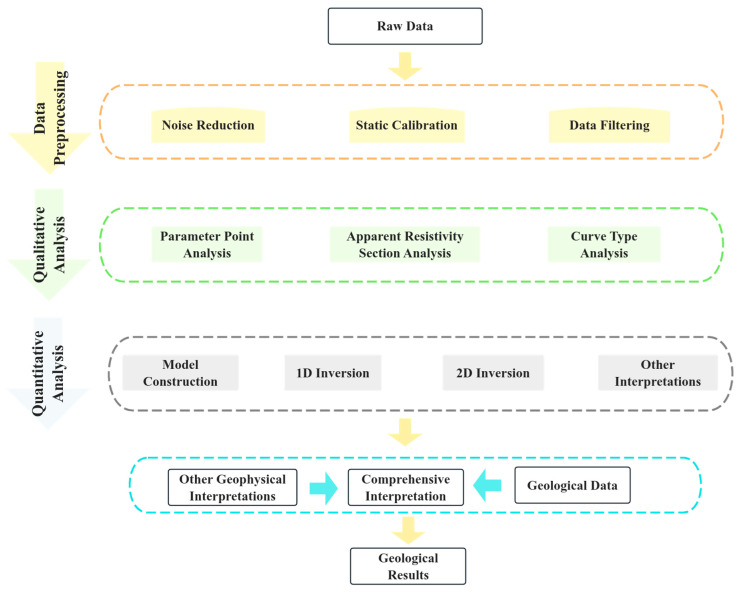
GEM-2 Data Processing Workflow.

**Figure 7:**
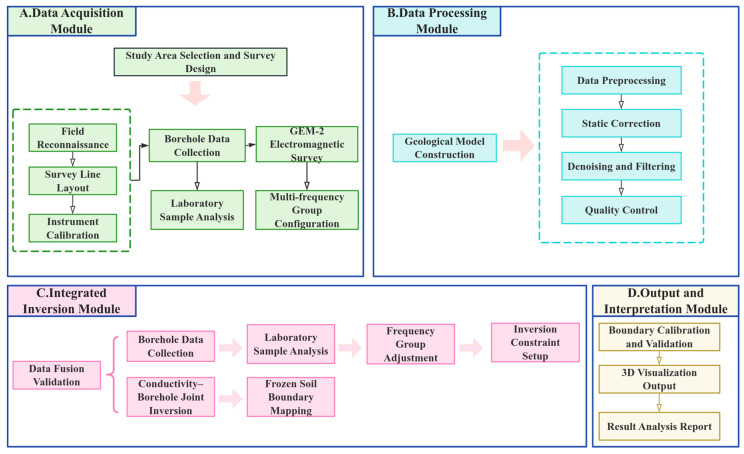
Processing workflow of the multi-frequency electromagnetic method for permafrost boundary detection.

Figure 6 presents the theoretical framework of the geophysical investigation, illustrating the conceptual basis for permafrost detection using multi-frequency electromagnetic (EM) methods. Building upon this framework, the comprehensive data processing and inversion workflow adopted in this study is summarized in Figure 7. It outlines the key stages from data acquisition, frequency selection and optimization, multi-frequency joint inversion, validation with borehole data, to the generation of final imaging outputs.

To transform the multi-frequency electromagnetic measurement data into physically meaningful subsurface conductivity structures, this study performed data processing and one-dimensional (1D) inversion using the Surfer software platform. The in-phase and quadrature responses recorded by the GEM-2 electromagnetic induction system at multiple frequencies were first converted into units of apparent conductivity (σ, in mS/m) using the GEM-2 Exporter tool and subsequently integrated with GPS spatial coordinates. The processed data was interpolated to generate two-dimensional (2D) conductivity profile images, illustrating the spatial distribution of electrical properties in the subsurface.

Given the typical stratigraphy of island permafrost regions—comprising an active layer, a permafrost body, and a weathered or saturated bedrock layer—this study employed a simplified 1D three-layer model for interpretation: as illustrated in Figure 5b, the upper layer corresponds to the active layer and weathered zone (relatively high conductivity), the intermediate layer represents the main permafrost body (marked decrease in conductivity), and the bottom layer reflects saturated bedrock or aquifers (increased conductivity). For each survey line, vertical apparent conductivity data were extracted at equal intervals, and a regular grid surface was constructed using Surfer’s “Grid from Data” module to produce 2D subsurface profile maps.

In this study, the permafrost boundary was operationally defined as the location of a sharp conductivity transition revealed by multi-frequency EM inversion, representing the shift from resistive permafrost to relatively more conductive strata. This “electrical boundary” serves as the electromagnetic induction (EMI)-equivalent proxy of the 0 °C isotherm, an approach that has been widely applied in EMI-based permafrost studies and provides a practical criterion for borehole validation .

During the inversion process, it was assumed that each observation point reflected a composite response dominated by a primary frequency. Different frequency combinations were used to construct “inter-layer contrast trend” images, enhancing the spatial resolution of permafrost boundary detection. For representing conductivity variations at different depths, the Kriging interpolation algorithm in Surfer was employed to reconstruct continuous apparent conductivity profiles, thereby revealing the electrical stratification patterns under various frequency combinations.

## 3. Results

### 3.1. Conductivity Responses Across Frequencies

**Figure 8:**
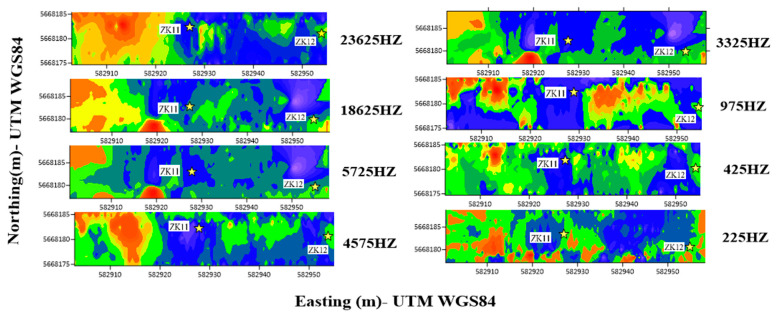
GEM-2 Conductivity Responses at Different Frequencies.

This field investigation utilized the GEM-2 multi-frequency electromagnetic induction instrument to assess conductivity response characteristics across different frequencies in a seasonally frozen transition zone. A representative survey line was established across an island permafrost area in the Greater Khingan Mountains, extending from borehole ZK11 in the permafrost zone (western segment) to borehole ZK12 in the non-permafrost zone (eastern end). As shown in Figure 8, the white labels indicate borehole names, while the yellow stars denote their schematic locations along the profile.

According to existing borehole records and regional geological surveys, the study area exhibits a typical three-layered electrical structure: an upper active layer, an intermediate permafrost layer, and a basal layer of weathered sandy bedrock. Electromagnetic responses vary significantly across these layers, particularly with regard to the spatial distribution of electrical conductivity.

Analysis of the 2D images (Figure 8) reveals a progressive increase in conductivity from ZK11 to ZK12 along the survey line. This trend is particularly pronounced at frequencies of 4575 Hz and 23,625 Hz. Apart from a few small-scale anomalies potentially induced by surface moisture or terrain disturbances, the trend remains consistent across all frequencies, suggesting a distinct contrast in electrical properties across the permafrost transition zone. Previous resistivity sounding studies in this area also identified zones of elevated conductivity concentrated within the permafrost boundary transition zone.

When comparing the responses across different frequency channels, high-frequency signals exhibit more prominent conductivity gradients near the inferred permafrost boundaries. This observation may be attributed to the enhanced sensitivity of high-frequency signals in high-resistivity media, combined with a skin depth that aligns more closely with the active layer–permafrost interface. These factors contribute to improved signal-to-noise ratios and enhanced boundary resolution [22]. Accordingly, this study recommends a “mid-to-high frequency combined” configuration strategy to simultaneously resolve shallow structural variations and capture the broader geometry of deep permafrost bodies.

### 3.2. Frequency Combination Evaluation

**Figure 9:**
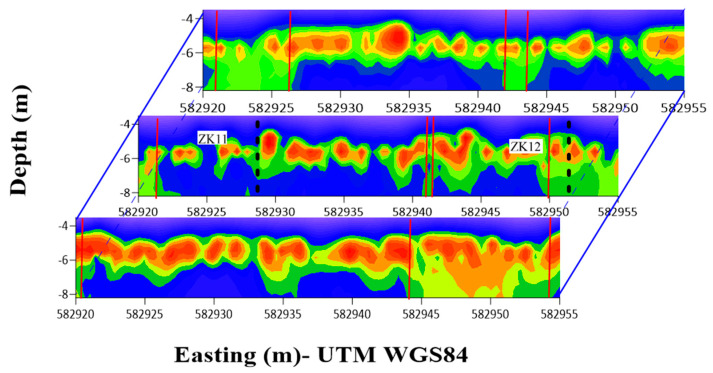
Three-Dimensional Resistivity Profile of the Freeze–Thaw Transition Zone.

Figure 9 presents a quasi-three-dimensional conductivity model constructed from the inversion results of three parallel survey lines within the study area, using a unified frequency combination (425 Hz, 975 Hz, 1575 Hz, 4975 Hz, and 23,625 Hz). The red vertical lines denote the permafrost boundary positions inferred from the joint multi-frequency inversion results. The central survey line covers the control section where borehole verification is available, whereas the two flanking lines are utilized to compare imaging consistency and frequency adaptability.

The inversion results reveal a distinct vertical stratification across all three lines: the shallow subsurface exhibits high conductivity and low resistivity, corresponding to the active layer and upper disturbed zone, while the deeper regions display a more stable distribution of low conductivity and high resistivity, indicative of the main permafrost body. On the central line, the red-marked boundary aligns well with a sharp conductivity gradient, exhibiting a clear demarcation. In contrast to conventional borehole techniques—which only provide discrete vertical temperature information at individual locations—the multi-frequency joint inversion employed in this study enables successful lateral delineation of the permafrost boundary across all three profiles.

Taking the central control line as an example, the multi-frequency EMI method significantly enhances the lateral continuity and spatial completeness of permafrost boundary delineation between boreholes, achieving a clearly defined and geophysically consistent interpretation. While the current sample size along this transect is insufficient for a full quantitative statistical analysis, the method demonstrates a clear improvement in revealing continuous boundary features compared to point-based borehole observations alone. This approach effectively reduces interpretation uncertainty in areas lacking direct borehole control and provides a robust basis for regional-scale boundary extrapolation.

In summary, the multi-frequency joint inversion strategy enables continuous and high-confidence identification of permafrost boundaries across all three survey lines. This approach substantially enhances the lateral extrapolation capability between sparse boreholes and offers a reliable technical pathway for delineating permafrost distribution in difficult-to-access regions, though further validation with additional boreholes would be required to establish comprehensive statistical metrics.

### 3.3. Sensitivity and Spatial Variability

The reliability of the multifrequency electromagnetic method for identifying permafrost boundaries was validated through analysis of two key segments along the survey line (582,925 E to 582,935 E and 582,950 E to 582,955 E, WGS UTM84). Joint interpretation of two-dimensional conductivity distribution maps and the 4975 Hz conductivity profile successfully extracted characteristic boundary points, demonstrating consistent spatial correspondence with borehole-verified permafrost interfaces and revealing underlying physical mechanisms through multi-data integration.

Around 582,930 m, the 2D conductivity image shows a distinct color transition zone, shifting sharply from a high-conductivity yellow area to lower-conductivity green and blue zones, indicating the presence of a significant electrical boundary. The corresponding 4975 Hz conductivity curve exhibits a pronounced peak, where the conductivity value surges from a background level of 4.19 mS/m to 22.1 mS/m before rapidly declining. The width of this transition zone is less than 0.8 m in the horizontal direction, demonstrating high lateral resolution. This electrical anomaly is likely associated with the permafrost boundary, presumed to be the interface between the active layer and the underlying permafrost. The localized conductivity enhancement may be attributed to the presence of unfrozen water or organic material within the −1 to 0 °C phase transition zone, leading to nonlinear increases in conductivity.

Further analysis of the 4575 Hz response reveals multiple high-conductivity zones between 582,925 m and 582,935 m, with maximum variations exceeding 15 mS/m. These anomalies suggest complex electrical structures and the presence of a wide thawing transition zone characterized by pronounced lateral heterogeneity. In contrast, the 582,950–582,955 m segment exhibits a generally smooth response, with minor conductivity fluctuations and isolated peak features only near the margins, indicating that this segment is situated within a thermally stable, non-permafrost zone.

**Figure 10:**
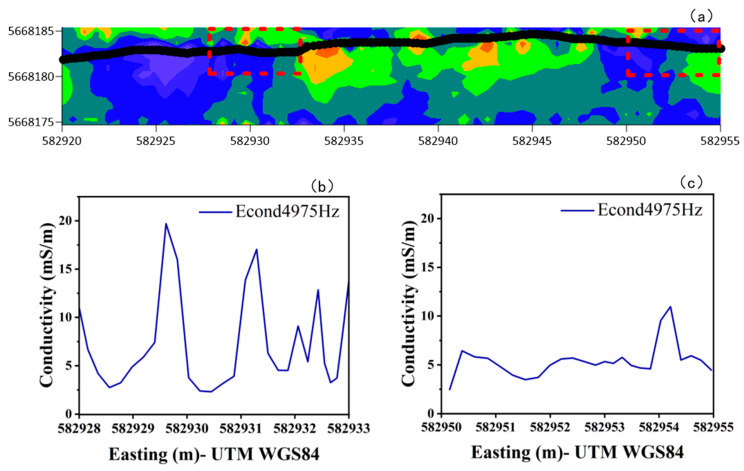
Two-Dimensional Apparent Conductivity Profile and Local Response Curves at 4975 Hz. (**a**) The 2D apparent conductivity distribution map along the corresponding survey line, where the black line represents the surface trace and the red dashed box highlights the key analysis area; (**b,c**) show the apparent conductivity variation curves at 4975 Hz corresponding to the left and right red box areas in (**a**), respectively.

As shown in Figure 10, the key boundary locations are identifiable across multiple frequencies, and the positions of abrupt changes are consistent between the 2D section maps and single-frequency response curves. This consistency verifies the effectiveness of the joint frequency–spatial analysis strategy in delineating permafrost boundaries. In particular, the 4575 Hz frequency demonstrates strong capability in resolving stratigraphic variations within the transition zone.

### 3.4. Optimized GEM-2 EM Process for Permafrost Boundary Delineation

To ensure data accuracy and interpretational reliability in delineating the boundaries of island permafrost using multifrequency electromagnetic (EM) methods, a comprehensive workflow has been established—encompassing data acquisition, preprocessing, inversion modeling, borehole validation, and final imaging output, as illustrated in Figure 7.

Prior to and following each field operation, static calibrations are conducted in zero-conductivity environments to obtain in-phase and quadrature offsets for each frequency channel under free-space conditions. These offsets are subsequently used for drift correction. During acquisition, the GEM-2 system records the responses of multiple frequencies and associated GPS coordinates synchronously at a sampling rate of 10 Hz.

Upon completion of field measurements, the preprocessing phase begins. This includes outlier removal to eliminate anomalous responses such as slip-induced spikes, signal saturation, or coil coupling imbalances. The data are then interpolated to a uniform 1 m spatial resolution, and linear drift correction is applied using static datasets recorded at the start and end of the survey window [23].

Following preprocessing, 1D inversions of the multifrequency response data are performed using Surfer software, based on a simplified three-layer subsurface model. In this model, the top layer represents the active layer or melt zone, the middle layer denotes the permafrost body, and the bottom layer is modeled as a homogeneous half-space (representing dry soil or stable frozen strata).

During parameter optimization, the inversion results are constrained and refined using borehole-derived information on stratigraphy, temperature profiles, moisture content, and ice saturation [24,25]. A pointwise inversion approach, combined with a sliding-window smoothing algorithm, is employed to enhance lateral continuity and minimize the influence of local anomalies on the overall interpretation.

For optimal frequency combination selection, sensitivity analyses and inversion error evaluations are performed to balance imaging depth with spatial resolution. The results are then validated against borehole data. If the inferred resistivity interface deviates significantly from the observed permafrost depth, parameter boundaries and initial inversion models are iteratively refined.

Upon successful integration and validation, the final permafrost boundary maps are generated by combining resistivity-derived interfaces with borehole depth calibrations. These are further subjected to three-dimensional visualization to intuitively reveal the spatial morphology of the permafrost layer.

This workflow not only enhances data accuracy but also establishes an integrated observation–inversion–validation–visualization framework, providing a robust technical foundation for non-invasive permafrost boundary mapping in complex terrains. Furthermore, it offers a practical reference for future survey design and frequency selection strategies across varying geological settings.

## 4. Discussion

Compared to previous permafrost detection methods, this study advances detection scale control, frequency configuration optimization, and boundary identification accuracy, demonstrating enhanced stability and adaptability in spatial electrical imaging within complex terrains of discontinuous permafrost zones.

Traditional permafrost boundary identification has primarily relied on three approaches: borehole [26], (high fidelity but costly and limited in coverage), electrical resistivity tomography (ERT) [27], (clear imaging but limited by electrode deployment), and single-frequency EM [28] (rapid but resolution-constrained). In contrast, frequency-domain electromagnetic methods offer non-contact operation, rapid deployment, and multi-frequency response capabilities, making them particularly suitable for identifying shallow-to-medium depth electrical discontinuity layers [23]. Our study employs the GEM-2 multi-frequency EM system to establish a detection framework with superior spatial coverage, resolution, and operational efficiency. This system enables continuous line acquisition in complex permafrost terrains without surface disturbance while maintaining excellent portability and field operability [29,30]. Its adaptability to diverse landscapes including hills, wetlands, and freeze–thaw transition zones [31,32,33]. significantly improves detection efficiency for discontinuous permafrost distribution.

Several methodological limitations and uncertainties warrant discussion. First, EM inversion is inherently constrained by physical principles and data sensitivity. Multi-frequency inversion results remain affected by subsurface heterogeneity [34], conductivity gradient transition zone width [35], and nonlinear induction depth effects [36], potentially causing discontinuous boundary identification or spatial fitting deviations in ice content gradients [32], pore connectivity, or groundwater disturbances [23]. Our simplified three-layer electrical model does not fully account for microstructural variations within frozen layers [37], such as ice content fluctuations or pore structure differences, which may cause blurred resistivity transitions in high-contrast zones [8,38].

Second, inherent ambiguity exists in the geophysical interpretation of permafrost boundaries. The GEM-2 system relies on resistivity responses, yet permafrost (high-resistivity bodies) and low-moisture bedrock formations exhibit similar EM characteristics [39], potentially leading to systematic misidentification. Furthermore, near-surface detection is highly sensitive to shallow heterogeneities—metallic debris, microtopography, and vegetation differences can all generate non-geological pseudo-boundary signals [25]. compromising boundary identification reliability. In this study, systematic diagnostic frequency-ratio tests or repeatability checks were not implemented due to field constraints. Instead, we qualitatively compared responses across multiple frequencies and employed borehole controls at ZK11 and ZK12 to cross-validate EM-inferred boundaries. These boreholes provided reliable reference points for distinguishing true permafrost boundaries from shallow artefacts or dry bedrock signals. Nevertheless, we acknowledge that dedicated repeatability experiments and quantitative frequency-ratio diagnostics would further strengthen the robustness of the method, and these will be incorporated into future work. These limitations necessitate calibration of GEM-2 inversion boundaries using direct observation methods like boreholes [26], where core temperature and ice content measurements can establish robust correlations between EM responses and permafrost states, thereby constraining inversion non-uniqueness and identifying external interference.

Additionally, as our data collection focused on the static freezing period, it does not capture the dynamic evolution of permafrost boundaries during seasonal freeze–thaw cycles, leaving temporal variation patterns incompletely resolved. Although the multi-frequency combination (e.g., 425 Hz, 975 Hz, 1575 Hz, 4975 Hz, and 23,625 Hz) demonstrated superior boundary identification performance in the quasi-three-dimensional resistivity model (Figure 9), the response relationship between frequency configuration and key permafrost physical properties remains to be systematically established. Future work could integrate forward modeling [40] and data-driven approaches [41] to deepen the physical understanding of frequency selection mechanisms and enhance the theoretical foundation of configuration strategies.

Finally, current permafrost boundary research still predominantly depends on borehole control point calibration [37,38]. While offering localized precision, this approach has limited investigation radius and cannot adequately capture regional-scale boundary variations. Moreover, borehole operations are time-consuming, costly, and sensitive to terrain and climate conditions, restricting their application in large-area continuous surveys. EM profiling improves overall detection efficiency and coverage. We recommend establishing a limited number of key boreholes in significant anomaly zones for model constraints and inversion accuracy control in future studies. Conversely, borehole-derived data (temperature, moisture content, and permafrost thickness) can inversely optimize EM inversion parameters, enhancing the system’s physical interpretability and stability. This complementary framework can substantially reduce reliance on high-density borehole networks, particularly valuable in logistically challenging, topographically complex, or budget-constrained permafrost regions [42]. By strategically configuring EM frequency combinations and optimizing borehole placement, this approach achieves an economical balance between “minimal boreholes for maximal precision,” offering significant engineering applicability.

In conclusion, while this study advances permafrost boundary identification in terms of scalability, precision, and efficiency, future work should incorporate time-series monitoring, 3D joint inversion, and multi-parameter coupled modeling to further improve method stability and regional adaptability. Such developments will provide more interpretative foundational support for permafrost degradation monitoring and engineering assessments.

## 5. Conclusions

This study employed the GEM-2 multi-frequency electromagnetic system to conduct high-resolution, non-contact boundary identification in a typical island permafrost region of the Greater Khingan Mountains, with multi-source cross-validation using borehole data. The main conclusions are as follows:

1. The GEM-2 multi-frequency electromagnetic system demonstrated strong environmental adaptability and engineering applicability in permafrost regions. Its features—rapid deployment, flexible frequency configuration, and stable signal response—make it particularly suitable for efficient surveys in complex terrains such as hilly areas, wetlands, and freeze–thaw transition zones.
2. The joint use of mid- and high-frequency channels significantly enhanced the ability to detect conductivity discontinuities in the shallow to intermediate subsurface. In the ZK11–ZK12 control section, the multi-frequency joint inversion achieved a continuous lateral delineation of the permafrost boundary, effectively bridging the ~23 m gap between boreholes. This represents a substantial improvement in spatial continuity over traditional interpolation methods that rely solely on sparse point data.
3. A cross-validation framework was established linking multi-frequency electromagnetic responses to inversion imaging, enabling a transition from qualitative interpretation to quantitative analysis of permafrost boundaries. This lays a solid foundation for subsequent model refinement and engineering application.
4. The inversion results exhibited strong spatial consistency with observed stratigraphy, electrical interfaces, and ground temperature profiles. The convergence of electrical discontinuities in key regions confirms the physical reliability and stability of the GEM-2 system in delineating permafrost boundaries.

In summary, the GEM-2 multi-frequency electromagnetic method offers significant potential for precise and efficient permafrost boundary detection. However, further optimization of frequency configuration strategies and consideration of internal heterogeneity within frozen soils are necessary to improve its applicability and detection accuracy, thereby providing a more robust technical tool for permafrost research and engineering practices.

## Data Availability

The datasets generated and analyzed during the current study are available from the corresponding author(s) on reasonable request.

## Abbreviations

The following abbreviations are used in this manuscript:

GEM-2	Geophysical Electro-Magnetic system, version 2
EM	Electromagnetic
SNR	Signal-to-Noise Ratio
